# Adolescent Psychotic Experiences and Adverse Mental Health Outcomes in Adulthood in a General Population Sample

**DOI:** 10.21203/rs.3.rs-4769284/v1

**Published:** 2024-08-14

**Authors:** Katrina M. Rodriguez, William W. Eaton, Russell L. Margolis, Keri Althoff, Rashelle J. Musci

## Abstract

Purpose This study estimated risk of incident mental disorders in adulthood associated with both transient and persistent adolescent psychotic experiences (PEs). Methods A nested case-control design was used within the Avon Longitudinal Study of Parents and Children (ALSPAC), a birth cohort study which recruited expectant mothers from 1991–1992. Participants consisted of 8822 offspring of ALSPAC mothers who completed the Psychosis-like Symptoms Interview Questionnaire (PLIKSi-Q). PEs were assessed using the PLIKSi-Q. Depressive disorders were assessed using the Short Mood and Feelings Questionnaire (SMFQ), anxiety disorders using the General Anxiety Disorder Assessment and the Clinical Interview Schedule-Revised, and psychotic disorder using the PLIKSi. Risk of incident depressive disorder, GAD, psychotic disorder, and past-year suicide attempts were compared amongst participants who had ever versus never reported a PE and those who reported persistent versus transient PEs. Results Adolescent PEs were associated with increased risk for incident depressive disorder (adjusted hazard ratio (aHR) = 1.62, 95% CI = 1.42, 1.84), GAD (aHR 1.23, 95% CI = 1.03, 1.47), psychotic disorder (adjusted odds ratio (aOR) = 5.08, 95% CI = 2.02, 12.79), and past-year suicide attempts (aHR = 2.56, 95% CI = 1.97, 3.25). Persistent PEs were associated with increased risk for depressive disorder (aHR = 1.81, 95% CI = 1.55, 2.12), generalized anxiety disorder (aHR = 1.34, 95% CI = 1.07, 1.68), and psychotic disorder (aOR = 7.39, 95% CI = 2.43, 22.19) but not past-year suicide attempts. Conclusion Adolescent PEs are a risk factor for multiple mental disorders and suicide attempts, with persistent PEs conferring greater risk. Identifying interventions for adolescents who report PEs, particularly persistent PEs, could lessen the burden of multiple mental health disorders and suicide attempts.

### Introduction

Psychotic experiences (PEs), characterized primarily by subclinical delusions and hallucinations, are often thought of as prodromal to full psychosis, however research shows that they are common in general populations, with cumulative incidence of 13.4% by age 24 years in a large population-based birth cohort study [1]. Lifetime prevalence of PEs in general population samples around the world are similarly high and are associated with multiple adverse mental health outcomes, but not deterministic [2, 3]. Current research shows PEs occurring in the absence of mental disorders increase the risk for current, lifetime, and comorbid mental disorders [4, 5]. A meta-analysis found that PEs in adolescence are associated with more than three times the risk of later developing any mental disorder [6]. However, when only the few longitudinal study designs available were included in the analyses, only the risk of psychotic disorders remained significant, leaving the question of whether other mental disorders share that longitudinal association. Additionally, later suicidal ideation, suicide attempts, and death by suicide are also increased in those who previously reported PEs [7–9]. These studies demonstrate the need for longitudinal studies examining the association of adolescent PEs and adult mental disorders.

Few studies examine persistent versus transient adolescent PEs. In the little research available, persistent PEs are associated with worse psychopathology. Researchers found increasing persistence of PEs over an eight-year period was associated with increased risk of clinical psychosis in a dose-response fashion [10]. Participants in the Helping to Enhance Adolescent Living Project who reported persistent PEs in adolescence were more than four times as likely to attempt suicide within a year compared to the no PE group, but those with transient PEs had no increased risk [11]. These studies provide evidence for increased psychopathology in late adolescence associated with persistent PEs, however longer-term follow-up studies into adulthood are needed.

Birth cohorts provide the ideal study design for examining these associations. Considering the overlap between early psychosocial and environmental risk factors for both PEs and mental disorders, accounting for these factors would greatly minimize residual confounding [1, 7, 10, 12–14]. The coming of age of birth cohorts has begun to provide more insights into long-term outcomes after PEs. For example, the Dunedin Multidisciplinary Health and Development study found that by age 38 years, participants with PEs at age 11 were 7.24 times more likely to be diagnosed with schizophrenia, 1.5 times more likely to be diagnosed with depression and anxiety, and 2.58 times more likely to die by or attempt suicide [15]. Within the Avon Longitudinal Study of Parents and Children (ALSPAC), PEs in adolescence were associated with psychotic disorder in early adulthood [1], and in the Mater-University of Queensland Study of Pregnancy (MUSP), persistent but (but not transient) hallucinations were associated with both psychotic disorders and suicide attempts by ages 30–33 years [16]. While not a birth cohort study, the Baltimore Epidemiologic Catchment Area (ECA) was one of the few to examine PEs' association with depressive disorders; in it, lifetime PEs were associated with incident depressive disorders in mid-to-late adulthood [17]. These studies add significantly to the epidemiologic evidence for the role of PEs in mental disorders, but the question remains whether adolescent PEs are a risk for other mental disorders and suicide attempts as well as psychotic disorders, and if so, whether the risk is greater in those with persistent versus transient PEs.

In this study, we compare incidence of multiple mental disorders and suicide attempts amongst those who reported PEs in adolescence in a historical birth cohort study followed prenatally until young adulthood [1]. This study comprises two aims; to separately compare the cumulative incidence of psychotic, anxiety, and depressive disorders and suicide attempts in young adulthood between participants who did and did not report PEs during adolescence and to compare the cumulative incidence of these disorders in young adulthood between those participants who reported persistent versus transitory PEs. We hypothesize that those with *any* adolescent PEs in the absence of mental disorders will have higher incidence of not only psychotic disorders, but also depressive and anxiety disorders and suicide attempts in adulthood compared to those who did not report adolescent PEs. We further hypothesize that those with persistent adolescent PEs will have higher incidence of these outcomes in adulthood compared to those with transient PEs.

### Methods

Participants were members of ALSPAC, a population-based birth cohort study that continuously follows offspring from the mother's pregnancy until the most recent data available at ages 24–28 years. Pregnant women in Bristol England and surrounding urban and rural areas with expected delivery dates between April 1991 and December 1992 were recruited to "investigate the modifiable influences on child health and development" [18, 19]. For more information on ALSPAC sampling methods, see Boyd, 2013, and Fraser, 2013, [18, 19]. Of the 14541 pregnancies enrolled, 13988 children were alive at one year. 913 additional children who were originally eligible for the study at conception but did not participate were recruited through various means between ages 7–24 years. For information on this subsequent recruitment see Northstone, 2019 [20]. This is a nested case-control study consisting of those participants who completed at least one PE questionnaire for comparisons between ever/never reported PEs and at least two PE questionnaires for comparisons between persistent/transient PEs. Details of all data are available through a searchable data dictionary and variable search tool on the study website (http://www.bristol.ac.uk/alspac/researchers/our-data/). Study data were managed and collected through REDCap at the University of Bristol [21]. Ethical approval for the study was obtained from the ALSPAC Law and Ethics committee and Local Research Ethics Committees (NHS Haydock REC: 10/H1010/70). Informed consent for the use of data collected via questionnaires and clinics was obtained from participants following the recommendations of the ALSPAC Ethics and Law Committee at the time.

## Psychotic Experiences

At ages 11, 13, 14, and 16 years participants completed the Psychosis-like Symptoms Interview Questionnaire (PLIKSi-Q), derived from the Diagnostic Interview Schedule for Children (DISC-IV) and the Schedules for Clinical Assessment in Neuropsychiatry (SCAN) which ascertain the presence of delusions and hallucinations that occurred in the past six months [22–24]. Participants were asked whether PEs occurred only in the presence of drugs, fever, or sleep-states to rule out misattribution. Sensitivity analyses were performed using structured clinical interviews administered at ages 12.5, 18, and 24 years [25]. PEs were considered present if they were endorsed as "definite" and not attributed to sleep, fever, or drugs. Transient PEs were defined as those endorsed at only one study visit and persistent PEs as those endorsed at two or more visits [26].

## Depressive Disorders

Depressive symptoms occurring over the previous two weeks were measured using the self-report Short Mood and Feelings Questionnaire (SMFQ) with scores greater than or equal to 12 considered depressed [27]. SMFQs administered at age 18 years (to control for prevalent disorders) and ages 21–25 years were used in main analyses.

## Anxiety Disorders

Both the Clinical Interview Schedule-Revised (CIS-R) and the Generalized Anxiety Disorder Assessment (GAD-7), a 7-item self-report scale with established validity and reliability, were used in analyses with generalized anxiety disorder (GAD) outcomes. The GAD-7 was administered at age 21 years and the CIS-R at ages 17–18 years and age 24 years [28, 29]. Participants with anxiety disorders at ages 17–18 years were excluded from analyses.

## Psychotic Disorders (probable)

Psychotic disorders were not explicitly measured in ALSPAC interviews. Therefore, we took the approach of previous research, classifying those who had definite PEs present once per month or more over the previous six months AND reported them as very distressing or causing a significant decline in social/occupational functioning OR led the participant to seek mental health treatment as a proxy for having psychotic disorder [30]. As in Zammit, 2013, the PLIKSi semi-structured interview at ages 17–18 years (to control for prevalent cases) and 24 years were used for outcome assessments [30].

## Suicide Attempts

Suicide attempts were assessed at 20-, 24-, and 28-years using self-report questionnaires based on the Child and Adolescent Self-Harm in Europe (CASE) study [31]. The participants were asked, "Have you ever hurt yourself on purpose in any way (e.g., by taking an overdose of pills, or by cutting yourself)?" Follow-up questions determined past-year frequency and suicidal intent. Those with suicidal intent at any assessment were classified as suicide attempts .

## Demographics

Participant's assigned sex at birth was included in analyses along with parental education (self-reported). Participants were more than 90% White non-Hispanic (self-reported), therefore race/ethnicity was not included in models.

## Covariates

Possible confounders for each outcome were determined by literature review followed by tests for significant differences between groups. Table 1 shows covariates included in analyses. Covariates spanned the prenatal period (i.e., maternal infection, substance abuse) through adolescence (i.e., adverse childhood experiences, including bullying, occurring prior to age 18). See Tables 2.1 and 2.2 in Supplement for all covariates considered. Covariates included in final analyses included prenatal anxiety, depression, substance use, and infection, pregnancy and birth complications, family history of mental disorders, childhood or adolescent trauma, including abuse, neglect, and bullying, childhood hyperactivity, emotional, conduct, or peer problems, prosocial behaviors, childhood emotionality and shyness, and oppositional defiant/conduct disorder (ODD/CD).

## Statistical Analysis

Continuous variables were tested for differences between groups using two-tailed Student's t-tests and categorical variables were tested using chi-squared tests.

Complementary log-log (clog-log) regression models estimating hazard ratios and 95% confidence intervals (CIs) were used to separately compare the cumulative incidence proportion of depressive and anxiety disorders and suicide attempts between those who reported PEs in adolescence and those who did not and between those who reported transient versus persistent PEs [32]. The time origin was defined as age 17–18 years, and participants with relevant outcomes at the time origin were excluded from analyses. Psychotic disorders (probable) were only assessed at one visit (24 years), therefore multivariate logistic regression was used. To account for missing data in the predictors and covariates, multiple imputation using chained equations (MICE) were used to create 25 imputed datasets [33].

Table 1
Comparison of "No Psychotic Experience (PE)" and "Any Psychotic Experience" and Persistent and Transient Psychotic Experience Groups by All Demographics and Covariates

| | **No PES (n = 5706)** | | **Any PEs (n = 3116)** | | |
|---|---|---|---|---|---|
| | N | (% / SE) | N | (% / SE) | p-value |
| Sex | | | | | |
| Female | 2838 | 60.4 | 1857 | 39.5 | **< 0.001** |
| Prenatal anxiety | 1008 | 19.3 | 709 | 25.2 | **< 0.001** |
| Prenatal depression | 894 | 17 | 576 | 20.3 | **< 0.001** |
| Infections during pregnancy | | | | | |
| Urinary tract infection *(any trimester)* | 641 | 11.9 | 380 | 13.1 | 0.114 |
| 1st trimester | 211 | 3.9 | 118 | 3.8 | 0.899 |
| 2nd and 3rd trimester | 480 | 8.9 | 300 | 10.3 | **0.034** |
| 3rd trimester | 236 | 4.4 | 163 | 5.6 | **0.012** |
| Thrush *(any trimester)* | 1174 | 20.6 | 690 | 22.2 | 0.081 |
| 1st trimester | 410 | 7.2 | 238 | 7.7 | 0.429 |
| 2nd and 3rd trimester | 961 | 16.9 | 5.88 | 18.9 | **0.016** |
| 3rd trimester | 529 | 9.3 | 337 | 10.9 | **0.019** |
| Other infection *(any trimester)* | 734 | 13.6 | 468 | 16.1 | **0.002** |
| 1st trimester | 245 | 4.5 | 167 | 5.7 | **0.016** |
| 2nd and 3rd trimester | 551 | 10.2 | 349 | 12 | **0.012** |
| 3rd trimester | 180 | 3.3 | 112 | 3.8 | 0.222 |
| Any infection *(any trimester)* | 2065 | 36.2 | 1221 | 39.2 | **0.005** |
| 1st trimester | 780 | 13.7 | 465 | 14.9 | 0.105 |
| 2nd and 3rd trimester | 1661 | 29.1 | 1024 | 32.9 | **< 0.001** |
| 3rd trimester | 841 | 14.7 | 551 | 17.7 | **< 0.001** |
| Substance use during mother's pregnancy | 217 | 4.1 | 177 | 6.1 | **< 0.001** |
| Family history of mental disorders (1st degree) | | | | | |
| depression | 34 | 6.7 | 248 | 8.8 | **0.001** |
| Birth complications | | | | | |
| pre-eclampsia | 231 | 7.4 | 175 | 9.2 | **0.023** |
| glycosuria | 159 | 2.9 | 126 | 4.3 | **0.001** |
| Childhood mental disorders | | | | | |
| ODD (Oppositional defiant or conduct disorders) | 103 | 2.3 | 88 | 3.6 | **0.002** |
| Childhood emotional and behavioral development | Mean Score | SE | Mean Score | SE | |
| hyperactivity | 1.98 | 0 | 2.23 | 0 | **< 0.001** |
| emotional problems | 0.95 | 0 | 1.17 | 0.1 | **< 0.001** |

| | No PES (n = 5706) | | Any PEs (n = 3116) | | |
|---|---|---|---|---|---|
| conduct problems | 0.87 | 0 | 1.02 | 0 | **< 0.001** |
| peer problems | 0.61 | 0 | 0.74 | 0 | **< 0.001** |
| total difficulties | 3.72 | 0.1 | 4.39 | 0.1 | **< 0.001** |
| Child temperament | Mean Score | SE | Mean Score | SE | |
| emotionality | 12.54 | 0 | 13.02 | 0.1 | **< 0.001** |
| activity | 20.35 | 0 | 20.48 | 0.1 | 0.082 |
| shyness | 11.93 | 0 | 11.56 | 0.1 | **< 0.001** |
| sociability | 18.17 | 0 | 18.26 | 0.1 | 0.161 |
| ACEs | 0.42 | 0 | 0.68 | 0 | **< 0.001** |
| | **TRANSIENT PES (n = 5706)** | | **PERSISTENT PEs (n = 3116 )** | | |
| | N | (% / SE) | N | (% / SE) | p-value |
| Sex | | | | | |
| Female | 1194 | 56.3 | 663 | 66.8 | **< 0.001** |
| Family history of mental disorders (1st degree) | | | | | |
| other psychopathology | 59 | 3.1 | 12 | 1.3 | **0.004** |
| Childhood emotional and behavioral development | Mean Score | SE | Mean Score | SE | |
| hyperactivity | 2.20 | 0.1 | 2.31 | 0.1 | 0.225 |
| emotional problems | 1.08 | 0 | 1.35 | 0.1 | **< 0.001** |
| conduct problems | 1.00 | 0 | 1.09 | 0 | 0.069 |
| peer problems | 0.67 | 0 | 0.85 | 0 | **< 0.001** |
| total difficulties | 4.19 | 0.1 | 4.81 | 0.2 | **0.002** |
| Child temperament | Mean Score | SE | Mean Score | SE | |
| emotionality | 12.91 | 0.1 | 13.25 | 0.1 | **0.018** |
| activity | 20.50 | 0.1 | 20.44 | 0.1 | 0.632 |
| shyness | 11.59 | 0.1 | 11.50 | 0.1 | 0.502 |
| sociability | 18.26 | 0.1 | 18.25 | 0.1 | 0.938 |
| ACEs | 0.59 | 0 | 0.89 | 0 | **< 0.001** |

## [Footnote] ACES = Adverse Childhood Experiences, SE = Standard Error

### Results

A total of 8822 participants completed a PLIKSi-Q at least once between ages 11 and 16 years, and 7282 at least twice. 8199 participants did not have any information on definite PEs and were excluded from analyses. Those lost to follow-up were less likely to report any PEs in adolescence (32.37% versus 36.9%, $p < 0.001$) and more likely to report transient versus persistent PEs (76.7% versus 64.2%, $p < 0.001$). They were also less likely to be female (39.18% versus 61.2%, $p < 0.001$) and had parents with a lower socioeconomic status (p = < 0.001). Mothers of those who were lost to follow-up were more likely to have reported substance use during pregnancy (6.7% versus 4.6%, $p < 0.001$) and to have experienced prenatal anxiety and depression (25.5% versus 21.3%, $p < 0.001$ and 24% versus 17.2%, $p < 0.001$, respectively). Of the 8822 participants who completed a PLIKSi-Q at least once, a total of 3116 (35.3%) participants reported at least one definite PE. Of the participants with a definite PE who completed the PLIKSi-Q two or more times (n = 2731), most reported transient PEs (n = 1736, 63.6%), 171 (6.3%) participants reported PEs at two times points, 259 (9.5%) at 3 times points, and 565 (20.6%) at all timepoints.

Compared to adolescents who did not endorse PEs, those who did were more likely to be male, have mothers who reported substance use in pregnancy, had prenatal complications such as depression, anxiety, infections (particularly after the second trimester), glycosuria, and preeclampsia. They were also more likely to have a family history of depression, greater emotional and behavioral problems in childhood, meet criteria for ODD/CD, and to report more ACEs. These differences were not present in those who reported persistent versus transient PEs except for specific childhood emotional/behavioral difficulties (peer and emotional difficulties) and increased ACEs. Those with persistent PEs were more likely to be female compared to those with transient PEs. (Table 1 **and Supplement Tables 2.1 and 2.2)**

## Psychotic Experiences and Depression

A total of 1360 participants were excluded due to meeting criteria for depression at baseline and a further 9659 participants were excluded due to missing data on depression at baseline, leaving n = 5784 for analyses. All results reported below are after adjustment. Those who reported any PEs in adolescence had greater risk of incident depression in young adulthood (aHR 1.62, 95% CI; 1.42, 1.84). There was higher risk of incident depression in young adulthood between those who reported persistent versus transient PEs (aHR 1.81, 95% CI; 1.55, 2.12). Results are shown in Table 2. Sensitivity analyses were performed examining only SMFQ and only CIS-R depression outcomes, neither of which substantially changed the results.

## Psychotic Experiences and Anxiety Disorders

A total of 257 participants were excluded due to meeting criteria for anxiety disorder at baseline and a further 10928 participants were excluded due to missing data on anxiety at baseline, leaving n = 4515 for analyses. Participants who reported any PEs were more likely to be diagnosed with incident GAD in young adulthood (aHR 1.23, 95% CI; 1.03, 1.47). Those who reported persistent versus transient PEs had higher risk of incident GAD (aHR: 1.34, 95% CI; 1.07, 1.68). There were no major changes to results when models were run using only the GAD-7 or only the CIS-R as outcome measures for anxiety.

## Psychotic Experiences and Psychotic Disorders (probable)

Participants without information on definite PEs were excluded from all analyses (n = 6621). A total of 40 participants were excluded due to meeting criteria for probable psychotic disorder at baseline, leaving n = 8782 for analyses. Participants who reported any PEs in adolescence had over 5 times the odds of incident psychotic disorder at age 24 years compared to those who did not (aOR 5.08, 95% CI; 2.01, 12.79). Those with persistent PEs had over 7 times the odds of incident psychotic disorder at age 24 years compared to those with transient PEs (aOR 7.39, 95% CI; 2.43, 22.19).

## Psychotic Experiences and Suicide Attempts

A total of 294 participants were excluded due to past-year suicide attempts at baseline and a further 10419 participants were excluded due to missing data on suicide attempts at baseline, leaving n = 4730 for analyses. Participants who reported any PEs in adolescence had increased risk of suicide attempts compared to participants who had no PEs (aHR 2.56, 95% CI; 1.97, 3.25). There were no differences between those who reported persistent versus transient PEs (aHR: 1.11, 95% CI; 0.80, 1.40).

For all analyses, using structured clinical interviews of the PLIKSi as the main predictor did not change the results; effect sizes were in the same direction, though generally much larger and more variable. Multiple imputation did not significantly change the results compared to complete case analysis for all analyses.

Table 2
Crude and Adjusted Hazard Ratios (HR) and 95% Confidence Intervals (CIs) for Mental Disorders and Past-Year Suicide Attempts

| **Hazard of mental disorders among those with (vs. without) psychotic experiences in adolescence** | | |
|---|---|---|
| | HR* (95% CI) | aHR* (95% CI) |
| Generalized anxiety disorder | **1.89 (1.63–2.20)** | **1.23 (1.03–1.47)** |
| Depressive disorder | **1.95 (1.78–2.14)** | **1.62 (1.42–1.84)** |
| Psychotic disorder** | **7.30 (3.19–16.66)** | **5.08 (2.02–12.79)** |
| Suicide attempts (past-year) | **2.92 (2.26–3.78)** | **2.56 (1.97–3.25)** |
| Hazard of mental disorders among those with persistent vs. transient psychotic experiences in adolescence | | |
| | HR (95% CI) | aHR (95% CI) |
| Generalized anxiety disorder | **2.24 (1.87–2.70)** | **1.34 (1.07–1.68)** |
| Depressive disorder | **2.22 (1.99–2.50)** | **1.81 (1.55–2.12)** |
| Psychotic disorder** | **1.49 (0.66 – 2.31)** | **7.39 (2.43–22.19)** |
| Suicide attempts (past-year) | 1.20 (0.88–1.65) | 1.11 (0.80–1.40) |

[Footnote] **Bold** *denotes significance at p < 0.05*, **bold italics** *denotes significance at p < 0.01*

## *HR = Hazard Ratio, aHR = Adjusted Hazard Ratio

***All outcomes except for psychotic disorder were analyzed using complementary log-log regression models due to psychotic disorder only measured at age 24. Psychotic disorder was analyzed using multivariable logistic regression.*

### Discussion

To our knowledge, this is the first study that compares incidence of mental disorders in young adulthood between those with persistent versus transient PEs in adolescence with PEs measured at four separate ages in adolescence. It is also the first to include a wide array of possible confounders from the neonatal stage through adolescence. In our examination of the association of adolescent PEs and incident mental disorders and suicide attempts in adulthood, PEs increased risk for depressive, anxiety, and psychotic disorders, and suicide attempts. We found PEs to increase risk of multiple other mental disorders and suicide attempts in addition to psychotic disorders. This study also shows the persistence of adolescent PEs to contribute to even greater risk of adult mental disorders than transient adolescent PEs, but that even a singular PE is impactful to future mental health. These results show that PEs, particularly persistent PEs, may be indicative of psychological distress and may represent an important intervention point in the etiology of multiple mental disorders or psychopathology in general.

The association of PEs and anxiety disorder has rarely been studied, with mixed results and little research on persistent PEs and anxiety. Our finding of adolescent PEs as a risk factor for later anxiety disorder is similar to findings in a birth cohort study by Poulton et. al., which reported an increased risk of anxiety disorders at age 26 years for those who reported PEs at age 11 years [34]. However, multiple other birth cohort studies did not find any association in PEs at 11 years and incident anxiety disorders [4, 15]. This could be because these discrepant studies used a stricter definition of anxiety disorders and/or may have underestimated the exposure due to only measuring PEs once. The MUSP study found that persistent hallucinations in late adolescence and early adulthood were not associated with anxiety disorders in mid-adulthood [16]. However, many anxiety disorders have early ages of onset and therefore the authors may have underestimated the association. The MUSP study also only measured hallucinations, which may be less a less salient risk factor for anxiety compared to delusions or a combination of PEs.

While we found adolescent PEs to be associated with incident depression between ages 20–26 years, previous research had mixed results. The Dunedin study found no increased risk for depression by age 38 years in those who reported PEs at age 11

years [15, 34]. The Adolescent Brain and Development Study ascertained PEs at age 11–12 years and adult mental disorders and found those in the PE groups did have a significantly higher percentage of depressive disorders at age 20–22 years compared to controls [4]. Our group previously found increased risk for incident depressive disorders in those with lifetime PEs in a 15-year follow-up of the Baltimore ECA [17]. The discrepancy in the Dunedin study could be due to an underestimate of the exposure by only measuring it at age 11 years or to that study's stricter criteria for depression, since our study used the SMFQ and the Dunedin studies used the DIS, with the additional criteria that the diagnosis occurred at least twice by age 38 years. As with anxiety disorders, we also found an increased risk for depressive disorders in those with persistent versus transient psychotic disorders. As the previous studies with depression as an outcome did not assess persistent PEs, we are unable to compare these results.

Our largest effect sizes were unsurprisingly for the association of PEs and psychotic disorders, both for ever/never and persistent/transient PEs. This is consistent with robust evidence available in the literature. In one of the first longitudinal analyses of PEs in adolescence and mental disorders, it was found that participants who reported PEs at age 11 years had more than 16 times the odds of a schizophreniform diagnosis by age 38 years [15]. A meta-analysis of PEs in childhood and adolescence and later mental disorders in community samples also found those with PEs had four times the risk of psychotic disorders in adulthood. Persistent PEs showed increased risk; the MUSP study found those with persistent hallucinations had over eight times the odds of psychotic disorders by mid-adulthood [6, 16].

Our finding that individuals reporting PEs in adolescence were more likely to attempt suicide in young adulthood is well-supported. One prospective cohort study found that increasing numbers of PEs predicted suicide attempts in a dose-response fashion [35]. However, a meta-analysis found PEs to be indicative of increased psychopathology, not specific to suicide attempts [36]. We did not find any significant differences in suicide attempts in young adulthood comparing persistent to transient PEs. However, another study found those with persistent, but not transient adolescent PEs, increased risk for suicide attempts at one year follow-up [11]. Previous research also reported persistent but not transient hallucinations to be associated with more than seven times increased risk of lifetime suicide attempts by age 30–33 years [16]. It is possible that the use of pooled logistic regression and much longer follow-up period increased power to detect differences. Additionally, this study used past-year suicide attempts, as opposed to lifetime suicide attempts; this would have missed any suicide attempts not measured in the three years for which we have data. Finally, we included delusions in our study which may have attenuated the results if hallucinations specifically are a risk factor for suicide attempts.

These results should be considered in combination with multiple limitations. Study participants were more than 90% White with less than 10% reporting low household income, limiting generalizability. The prevalence of PEs during adolescence may have been overestimated since we used questionnaire responses as opposed to structured clinical interviews; however, in sensitivity analyses using only structured clinical interview assessments of PEs our results remained significant. There is research suggesting PEs are over-estimated and may be a symptom of anxiety and not true psychotic experiences; this would require a trained clinician to assess the validity of PEs, which is beyond the scope of this study [37]. Additionally, the PLIKSi ascertains PEs over the past six months; this could mean a participant had multiple PEs over a 6-month period yet was only marked as "transient" if these PEs were only captured at the last or at one timepoint. However, by including multiple timepoints, the majority of those with persistent PEs were likely captured. Since we included up to age 16 years in the exposure variable, it is possible we missed more severe disorders which typically have earlier ages of onset. Another limitation is in our use of a proxy variable for psychotic disorders. The criterion for psychotic disorders used is broad and may include participants who would not otherwise meet criteria for the disorder by DSM or ICD standards, thereby possibly overestimating our results. However, our effect sizes for psychotic disorders, while large, are not inconsistent with previous literature.

This study also has multiple strengths. PEs and mental disorders and suicide attempts in adulthood share many risk factors, including prenatal infections, prenatal anxiety and depression, obstetric complications, and childhood adversity and disorders [1, 7, 13, 14, 38, 39]. Our study is unique in its inclusion of such a wide range of possible confounding variables occurring from prenatal stages through childhood. The longitudinal design of birth cohort studies allows causal inferences to be made, minimizes misclassification error of PE categories, minimizes recall bias, and captures the main periods of mental disorder onset. We used a variety of sensitivity analyses, including only using semi-structured interviews which are less likely to overestimate PEs compared to self-reports, and our results were unchanged, increasing confidence in our results. Additionally, we ruled out cases of PEs that may have been due to sleep, fever, or drugs, and only included those endorsed as "definite". The capture of PEs throughout adolescence enabled us to form a better understanding of the relationship between adolescent PEs and adult mental disorders.

### Conclusion

As a 2019 meta-analysis and systematic review pointed out, there is a need for more research on the relationship between childhood PEs and later mental disorders [6]. With the coming of age of ALSPAC participants, we were able to show that PEs increased the risk for anxiety disorders, depressive disorders, psychotic disorders, and suicide attempts. This suggests that transient PEs are an important intervention point and those with persistent PEs are especially vulnerable to later mental disorders. Future research should examine specific PEs' association with mental disorders and expand on this research as birth cohorts age.

### Declarations

## Funding:

The UK Medical Research Council and Wellcome (Grant ref: 217065/Z/19/Z) This research was specifically funded by the following grants: 076467/Z/05/Z, MR/L022206/1, MR/M006727/1, 08426812/Z/07/Z, G0800612/86812, 5R01MH073842- 04, 61356, MR/M006727/1, 102215/2/13/2, 102215/2/13/2, 1215–20011, 102215/2/13/2, MR/M006727/1, PD301198- SC101645, G0701594, 092731, 5R01AA018333-0, RP-PG-0108-10048, PR-RS-0912-11023, 5R01AA018333-05, 092731, 092731, GR067797MA, and WT092830/Z/10/Z. Dr. Katrina M. Rodriguez was supported by 5T32MH14592-45, PI: Dr. Heather Volk, and 5T32MH020004-24, PI: Dr. Patrick Brown.

## Author Contribution

Conceptualization: Katrina M. Rodriguez, MHS, PhD, William W. Eaton, PhD, PhD, Rashelle J. Musci, PhD; Methodology: Katrina M. Rodriguez, MHS, PhD, William W. Eaton, PhD, Keri Althoff, PhD, Rashelle J. Musci, PhD; Formal analysis and investigation: Katrina M. Rodriguez, Rashelle J. Musci, PhD ; Writing - original draft preparation: Katrina M. Rodriguez, MHS, PhD, William W. Eaton, PhD, Russell Margolis, MD, Keri Althoff, PhD, Rashelle J. Musci, PhD; Writing - review and editing: Katrina M. Rodriguez, MHS, PhD, William W. Eaton, PhD, Russell Margolis, MD, Keri Althoff, PhD, Rashelle J. Musci, PhD; Supervision: William W. Eaton, PhD, Rashelle J. Musci, PhD

## Acknowledgement

The study authors are grateful to the families who participated in the ALSPAC study, the midwives who helped recruit them, the entire ALSPAC team, including interviewers, computer and laboratory technicians, clerical workers, research scientists, volunteers, managers, receptionists, and nurses.

## Data Availability

Details of all data are available through a searchable data dictionary and variable search tool on the study website (http://www.bristol.ac.uk/alspac/researchers/our-data/).

### Supplementary Files

This is a list of supplementary files associated with this preprint. Click to download.

- AdolescentPsychoticExperiencesandAdverseMentalHealthOutcomesinAdulthoodinaGeneralPopulationSampleappendix.docx
